# High Hydrostatic Pressure Pretreatment of Whey Protein Isolates Improves Their Digestibility and Antioxidant Capacity

**DOI:** 10.3390/foods4020184

**Published:** 2015-05-28

**Authors:** Michèle M. Iskandar, Larry C. Lands, Kebba Sabally, Behnam Azadi, Brian Meehan, Nadir Mawji, Cameron D. Skinner, Stan Kubow

**Affiliations:** 1 School of Dietetics and Human Nutrition, McGill University, 21,111 Lakeshore, Ste. Anne de Bellevue, QC H9X 3V9, Canada; E-Mails: michele.iskandar@mail.mcgill.ca (M.M.I.); kebba.sabally@mcgill.ca (K.S.); Behnam.azadi@mcgill.ca (B.A.); 2 Montreal Children’s Hospital – McGill University Health Centre, Division of Pediatric Respiratory Medicine, Room D380, 2300 Tupper Street, Montreal, QC H3H 1P3, Canada; E-Mails: larry.lands@muhc.mcgill.ca (L.C.L.); bmeehan82@hotmail.com (B.A.); nadir.mawji@mail.mcgill.ca (N.M.); 3 Department of Chemistry and Biochemistry, Concordia University, 7141 Sherbrooke Street W., Montreal, QC H4B 1R6 Canada; E-Mail: CSkinner@alcor.concordia.ca

**Keywords:** pressurized whey, digestibility, antioxidant, anti-inflammatory

## Abstract

Whey proteins have well-established antioxidant and anti-inflammatory bioactivities. High hydrostatic pressure processing of whey protein isolates increases their *in vitro* digestibility resulting in enhanced antioxidant and anti-inflammatory effects. This study compared the effects of different digestion protocols on the digestibility of pressurized (pWPI) and native (nWPI) whey protein isolates and the antioxidant and anti-inflammatory properties of the hydrolysates. The pepsin-pancreatin digestion protocol was modified to better simulate human digestion by adjusting temperature and pH conditions, incubation times, enzymes utilized, enzyme-to-substrate ratio and ultrafiltration membrane molecular weight cut-off. pWPI showed a significantly greater proteolysis rate and rate of peptide appearance regardless of digestion protocol. Both digestion methods generated a greater relative abundance of eluting peptides and the appearance of new peptide peaks in association with pWPI digestion in comparison to nWPI hydrolysates. Hydrolysates of pWPI from both digestion conditions showed enhanced ferric-reducing antioxidant power relative to nWPI hydrolysates. Likewise, pWPI hydrolysates from both digestion protocols showed similar enhanced antioxidant and anti-inflammatory effects in a respiratory epithelial cell line as compared to nWPI hydrolysates. These findings indicate that regardless of considerable variations of *in vitro* digestion protocols, pressurization of WPI leads to more efficient digestion that improves its antioxidant and anti-inflammatory properties.

## 1. Introduction

Whey proteins have a high biological value due to their high content of indispensable amino acids and are widely used by athletes to reduce protein degradation and muscle loss during heavy exercise [1,2]. In recent years, a number of biological activities have been attributed to whey proteins beyond their nutritional value making them the subject of increasing interest towards functional food use. Whey protein digestion generates bioactive peptides of varying molecular weights that are absorbed systemically via transcellular and paracellular processes [3] to exert biofunctional effects including antimicrobial, antiviral, anti-carcinogenic, antioxidant, opioid and angiotensin converting enzyme (ACE)-inhibitory activities [4,5,6,7,8,9], immunomodulatory effects [10,11,12,13] and can decrease blood pressure and serum lipid levels [14]. Enzymatic hydrolysis of proteins is often used to produce hydrolysates with functional or bioactive properties and *in vitro* digestion of whey proteins has been utilized to identify bioactive peptides with antioxidant [15], anti-inflammatory [16] and ACE-inhibitory properties [17]. 

High hydrostatic pressure treatment can disrupt protein secondary, tertiary and quaternary structures and alter their conformation, thereby exposing otherwise hidden peptide sequences to proteolytic cleavage [18]. This can enhance their digestibility and the bioavailability of peptides derived from their enzymatic hydrolysis and may potentiate the bioactive properties of such hydrolysates. Research involving the enzymatic digestion of proteins under high hydrostatic pressure has shown accelerated reaction rates that were attributed to the conformational changes undergone by the substrate rather than pressure-induced increases in enzymatic activity [19,20,21]. However, in the context of whey proteins as functional foods, the effect of pressure-induced conformation changes on subsequent digestibility by human gastrointestinal enzymes is more relevant. In that regard, animal studies have shown that pressurization of whey protein isolates (pWPI) prior to feeding potentiates its tissue glutathione (GSH)-enhancing [22] and antibacterial [4] effects as opposed to native whey protein isolates (nWPI). In human studies, supplementation with pWPI led to a dose-response increase in lymphocyte GSH levels [23], improved nutritional status and markers of systemic inflammation in patients with cystic fibrosis [24], and increased cycling endurance test time in patients with chronic lung disease [25].

*In vitro* enzymatic hydrolysis of proteins is used to explore the bioactive properties of the peptides produced. A previous study [26] on the effects of *in vitro* digestion of pWPI demonstrated altered peptide profiles and enhanced anti-inflammatory properties of the resulting peptides. However, the choice of enzymes, conditions, enzyme:substrate (E:S) ratios and membrane filter pore size to collect the resulting peptides was quite dissimilar from *in vivo* digestion. Furthermore, variations in digestion protocol parameters have been shown to result in whey protein hydrolysates with markedly different peptide profiles and biological activities [27,28]. 

The current study was undertaken to study the digestibility and peptide profiles of native whey protein isolates (nWPI) and pWPI via an *in vitro* digestion system designed to better mimic human digestion. We have previously shown that high hydrostatic pressure pretreatment of WPI enhanced the anti-inflammatory and antioxidant activities of whey protein hydrolysates in respiratory epithelial cells exposed to lipopolysaccharides (LPS) [29]. We therefore investigated whether major variations in *in vitro* digestion parameters can alter the enhanced antioxidant and anti-inflammatory effects of hydrolysates from pressure-processed WPI.

## 2. Materials and Methods

### 2.1. Materials

Inpro 90 WPI was purchased from Vitalus (Abbotsford, BC), with the following composition: protein (dry basis) ≥92%; β-lactoglobulin (β-LG) 43%–48%; GMP 24%–28%; α-lactalbumin 14%–18%; bovine serum albumin (BSA) 1%–2%; immunoglobulins 1%−3%; lactoferrin <1%. Pepsin from porcine stomach mucosa, porcine pancreatic trypsin, bovine pancreatic chymotrypsin, porcine intestinal peptidase, pancreatin from porcine pancreas, and *O*-phthalaldehyde (OPA), were purchased from Sigma-Aldrich. Amicon regenerated cellulose ultrafiltration membranes of Molecular Weight Cut-Off (MWCO) 1 and 10 kDalton (kDa) and ultrafiltration stirred units were purchased from Millipore. Bradford reagent was purchased from BD Biosciences. Ferric chloride was purchased from ACP Chemicals Inc., l-ascorbic acid was bought from Fisher Scientific. Sodium acetate trihydrate, glacial acetic acid and 2,4,6-tri(2-pyridyl)-s-triazine (TPTZ) were purchased from Sigma-Aldrich. All other chemicals were purchased from Sigma-Aldrich and were of highest analytical grade.

### 2.2. Hyperbaric Treatment of WPI

The WPI was dissolved (15% solution) in double-distilled water (ddH_2_O) and pressurized with an Avure High Pressure Processing System model QFP 215L-600 (Avure Technologies, Columbus, OH, USA). As pressures above 500 MPa are required to denature most whey proteins [30], one cycle of pressurization at 550 MPa at 20 °C was carried out. The pressure indicated was achieved within 3–4 min, followed by 1 min holding time and depressurization took place in under 30 sec. At least three separate treatments were performed. Control native WPI (nWPI) underwent the same treatment with omission of the pressurization step. The solutions were then frozen overnight at −80 °C and immediately freeze-dried and stored at −20 °C under nitrogen gas until further use.

### 2.3. In Vitro Enzymatic Digestion and Peptide Isolation

Lyophilized pWPI and nWPI samples were dissolved in ddH_2_O at a concentration of 3 mg/mL and at 37 °C. The pH was adjusted to 1.9 with addition of 1 N or 10 N HCl. First-stage digestion was performed with pepsin (prepared in 0.01 M HCl; E:S ratio 1:200) for 15 min, after which the pH was adjusted to 7.4 with addition of 10 N NaOH. Second-stage digestion was performed with trypsin, chymotrypsin and peptidase (prepared in phosphate buffer pH 7.0, E:S ratios 1:200, 1:87, and 1:120 respectively) for 60 min, after which the enzymes were inactivated with the addition of 10 N NaOH (pH 10.5). Immediately upon inactivation of the proteolytic enzymes, the entire mixture was chilled on ice and subjected to ultrafiltration. Briefly, to remove high molecular weight peptides, a membrane filter with a MWCO of 10 kDa (Millipore, Nepean, ON, USA) was used in a stirred ultrafiltration membrane reactor (Amicon Ultrafiltration Cell, model 8050) at 4 °C and under nitrogen gas pressure of 40 psi. The filtrates were freeze-dried and stored at −80 °C under nitrogen gas until further use. The resulting hydrolysates are termed pressurized whey protein hydrolysates (pWPH) and native whey protein hydrolysates (nWPH). In addition, hydrolysates of pressurized WPI were also prepared according to the digestion protocol (pWPB) previously described by Vilela *et al.* [26]. Briefly, pWPI was dissolved in ddH_2_O at a concentration of 3 mg/mL and at 37 °C. First stage digestion was performed with pepsin at an E:S ratio of 1:100, pH 1.5, for 30 min. Second stage digestion was performed with pancreatin at an E:S ratio of 1:30, pH 7.8, 40 °C, for 60 min, following which the reaction was stopped with the addition of 150 mM Na_2_CO_3_. Ultrafiltration was performed using a membrane filter of MWCO of 1 kDa.

The modifications applied to the previous digestion protocol of Vilela *et al.* [26] are as follows:

*Temperature*. Incubation temperatures were previously chosen for optimal activities of the digestive enzymes (37 °C and 40 °C for pepsin and pancreatin, respectively). In the present study, temperature was maintained at 37 °C throughout the entire digestion procedure.

*pH*. The pH of the solution was maintained at 1.9 during pepsin digestion and at 7.4 during digestion with pancreatic enzymes (as opposed to pH 1.5 and 7.8) based on the work of Gauthier *et al.* [31], Agudelo *et al.* [32] and Qiao *et al.* [33]. Furthermore, since the digestion was carried out in a non-buffered solution, the drop in pH during pancreatic digestion due to protein hydrolysis was off-set by the continuous addition of 1 N NaOH to maintain a constant pH of 7.4.

*Incubation time*. The initial length of peptic digestion was set at 30 min, based on the gastric half-emptying time observed *in vivo* [34]. However, it has been observed that whey proteins, which do not coagulate under acidic conditions, remain soluble and exit the stomach more rapidly than caseins [35,36]. Therefore the length of peptic digestion was shortened to 15 min.

*Enzymes*. Pancreatin is a concentrated and lyophilized mixture of several enzymes including amylase, trypsin, ribonuclease, and lipase. However, the relative concentrations of the different enzymes within the mixture are undefined. Although it is possible to adjust the E:S for pancreatin *per se*, the actual amounts of individual proteolytic enzymes may vary. Batch to batch variability could also hinder the standardization of the digestion procedure. In order to control the concentrations of proteolytic enzymes used during the digestion, the proteases trypsin, chymotrypsin and aminopeptidase were obtained separately and added simultaneously during the second stage of digestion. Furthermore, aminopeptidase, a brush-border enzyme, was included as it is not found in pancreatin.

*Enzyme to substrate ratio (E:S)*. For pepsin, the E:S was decreased from 1:100 to 1:200. Rat feeding experiments have suggested that, prior to reaching the intestine for further hydrolysis, about 30% of ingested protein is hydrolyzed in the stomach [37]. In an in-depth study of different enzymatic conditions for *in vitro* protein digestion, Gauthier *et al.* [31] have shown that a lower E:S ratio for peptic hydrolysis allows for the production of approximately 30% of trichloroacetic acid (TCA)-soluble nitrogen after 15–30 min. In addition, the lower E:S ratio reduces the amount of amino acid contamination from pepsin hydrolyzed by subsequent digestive enzymes [31]. Similar ratios for pepsin hydrolysis (1:200 to 1:250) have been used in most studies involving *in vitro* protein digestion [32,38,39]. The E:S ratios for the pancreatic enzymes were calculated based on the methodologies of Kent *et al.* [15], Wong and Cheung [40], and Hsu *et al.* [41].

*Ultrafiltration membrane molecular weight cut-off (MWCO)*. The MWCO of an ultrafiltration membrane is a nominal value expressed in Daltons (Da) and is defined by the ability of the membrane to retain at least 90% of a globular molecule of said molecular weight [42]. Therefore, in order to obtain a filtrate containing a maximal recovery of peptides of approximately 1000 Da in size, it is recommended by the manufacturer that a filtration membrane with a significantly greater MWCO than the size of the desired solute should be used. In that regard, ultrafiltration membranes with a MWCO of 10,000 Da were used in the current study, as opposed to 1000 Da.

### 2.4. Digestibility Assays

Digestibility was assessed as follows: samples were collected during the digestion procedure at 0, 5, 10 and 15 min of stage 1 (pepsin) and at 15, 30, 45 and 60 min of stage 2 (pancreatic enzymes). Protein content was assessed using the Bradford method [43]. Samples were incubated for 5 min with Bradford reagent at a 1:50 ratio and absorbances were read on a spectrophotometer at 590 nm. A standard curve was constructed using BSA as a standard. The degree of hydrolysis (DH) at different time points (Ti) was calculated as follows: DH = (protein content at Time 0—protein content at Ti)/(protein content at Time 0) × 100%. The α-amino group content was determined using the OPA reagent, according to the method of Church *et al.* [44], which measures primary amine groups in amino acids, peptides and proteins. The OPA reagent was prepared by adding 25 mL of 100 mM sodium tetraborate in water, 2.5 mL of 20% (wt/wt) sodium dodecyl sulfate, 40 mg of OPA dissolved in 1 mL of methanol, 100 μL of β-mercaptoethanol and ddH_2_O to a final volume of 50 mL. A 50 μL sample of digestion mixture was incubated for 2 min with 950 μL OPA reagent solution. Absorbances were read at 340 nm on a spectrophotometer. A standard curve was constructed using leucine-glycine as a standard peptide.

### 2.5. Characterization of Hydrolysate Peptide Profiles

The peptide profiles of lyophilized hydrolysates were analyzed by capillary zone electrophoresis (CZE) and Reverse-Phase High Performance Liquid Chromatography (RP-HPLC). CZE was carried out using a Beckman Coulter P/ACE MDQ capillary electrophoresis system (Fullerton, CA, USA). A fused-silica capillary (60 cm in length, window at 50 cm, 75 μm i.d., 360 μm o.d.) from Polymicro Technologies (Phoenix, AZ, USA) was conditioned in between runs by flushing for 1 min at 20 psi, followed by a 0.25-min wait at 0 bar with 5 mM SDS and then 100 mM sodium hydroxide. The capillary was filled with 50 mM phosphate buffer (pH 2.3; 2 min at 20 psi) and conditioned under 15 kV for 1 min (0 psi). Samples were injected hydrodynamically (0.5 psi, 5 s) and separated using 50 mM phosphate buffer (pH 2.3) with 15 kV at 28 °C. Electropherograms were generated at 190, 254, and 275 nm (5 nm bandwidth, 4 Hz) from the photodiode array. Peak integration of the resulting electropherograms was done using OriginPro v8.0. For HPLC, samples were analyzed using a Varian HPLC system with a tertiary gradient pump, a variable wavelength UV/VIS detector, and an autosampler with refrigerated sample compartment (Varian Canada Inc, Mississauga, ON, USA). Samples were eluted using an Onyx reverse-phase HPLC column (100 × 4.5 mm) (Phenomenex, CA, USA), using a solvent flow rate of 1 mL/min and detection was at 215 nm. Gradient elution was carried out with a mixture of two solvents. Solvent A: 0.05% trifluoroacetic acid (TFA) in 10% aqueous acetonitrile (ACN) and solvent B: 0.05%TFA in 60% aqueous ACN, (v/v) starting with 100% solvent A and reaching 40% solvent A and 60% solvent B in 30 min.

### 2.6. LC-ESI-TOF-MS Analysis of nWPH and pWPH

Electrospray time-of-flight mass spectrometry (ESI-TOF-MS) was carried out using an Agilent 1200 HPLC system equipped with an Agilent 6210 time-of-flight (ESI-TOF) mass spectrometer (Santa Clara, CA, USA). The nWPH and pWPH samples were separated using gradient conditions on an Agilent Eclipse C18 column (3 × 50 mm; 1.8 μm) (Agilent Technologies, CA, USA) heated to 60 °C. Elution was achieved using solvent A (water + 0.1% formic acid (FA)) and B (100% acetonitrile + 0.1% FA). Gradient conditions were: 5% B at 1 min to 45% B at 25 min, to 95% B at 25.5 min and 95% B at 28 min to 5% B at 28.1 min and 5% B at 32 min with a flow rate of 0.3 mL/min and 5 μL of sample was injected. Accurate mass data were obtained using a dual ESI source in both positive and negative mode (injected in two different methods): data was acquired over a mass range of m/z 100–1000. The source was operated with the following parameters: temperature 350 °C; gas flow 12 L/min; nebulizer 50 psi; capillary voltage 4000 V; fragmentor 100 V; skimmer voltage 60 V. Reference masses (internal calibration of high resolution spectra) were: positive mode: *m/z* 121.050873, 922.009798; negative mode: *m/z* 119.03632, 966.000725.

### 2.7. Antioxidant Capacity of Hydrolysates

The antioxidant capacity of the freeze-dried hydrolysates was assessed using the Ferric Reducing Antioxidant Power (FRAP), based on the reduction of the Fe^3+^-2,4,6-tripyridyl-*S*-triazine complex to the ferrous (Fe^2+^) form, performed according to the method of Benzie and Strain [45]. FRAP reagent was prepared with sodium acetate buffer (300 mM), 2.5 mL TPTZ solution (10 mM in 40 mM HCl), and 2.5 mL ferric chloride solution (20 mM in ddH_2_O) in a 10:1:1 ratio, respectively. After ultrafiltration, the concentration of peptides in mg/mL cannot be determined based on the initial protein concentration alone. Therefore, the filtrate was freeze-dried and reconstituted at 10 mg/mL for both native and pressurized for a more accurate comparison. Lyophilized reconstituted WPI hydrolysates were incubated with FRAP reagent for 90 min at a 1:30 ratio. A standard curve was constructed with BSA and absorbances were read at 593 nm.

### 2.8. Cell Culture and Experimental Studies

An immortalized human respiratory cell line utilized in this study, 1HAEo-, was a kind gift from Dr. D. Gruenert (University of California, San Francisco, CA, USA). We have previously described the basal cell culture conditions [29,46]. For interleukin-8 (IL-8) production experiments, cells were seeded at a density of 5 × 10^5^ cells/mL in 24-well cell culture plates and grown for 24 h in MEM supplemented with L-glutamine, penicillin/streptomycin, and 10% heat-inactivated FBS. The next day cells became 100% confluent and were pre-incubated for 1 h with pWPH or pWPB hydrolysates (0–1000 μg/mL) in antibiotic-free MEM supplemented with 2% FBS. Cells were then stimulated with 2.5 μg/mL lipopolysaccharide (LPS; Sigma-Aldrich) for 24 h, along with a fresh preparation of hydrolysates, following which IL-8 secretion in cell-free supernatants was assessed. 

### 2.9. Interleukin-8 Analysis

IL-8 secretion in cell-free supernatant was assessed with a commercial human IL-8 ELISA kit (BD Biosciences) according to the manufacturer’s instructions.

### 2.10. Analysis of Antioxidant Capacity of Cell-Free Supernatants

1HAEo- cells were incubated with pWPH or pWPB hydrolysates for 6 h, following which cell-free supernatants were collected. The antioxidant capacity of these supernatants was assessed using the FRAP assay [45].

### 2.11. Statistical Analysis

Data are presented as mean ± SEM. All experiments were performed in triplicate. For digestibility assays, results were compared by two-way ANOVA with Tukey’s post-hoc test for multiple comparisons. Differences in antioxidant capacities and in the CZE profiles percent peak areas between the nWPI and pWPI hydrolysates were analyzed using *t*-tests. A *p*-value < 0.05 was considered significant. Statistical analyses were performed using Sigma Stat v2.03 (Systat Software Inc., Chicago, IL, USA). 

## 3. Results

### 3.1. Effect of Hyperbaric Treatment on in Vitro Digestibility of Whey Proteins

**Figure 1:**
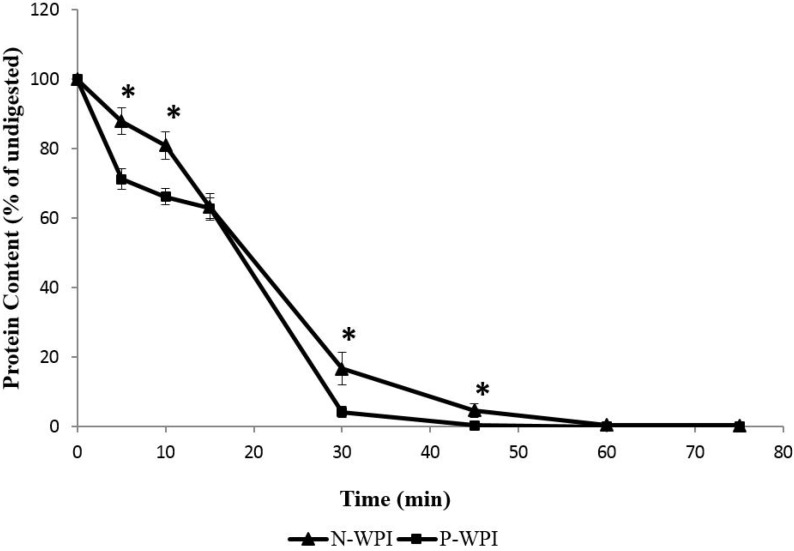
Effect of high hydrostatic pressure on the *in vitro* proteolysis of nWPI and pWPI. Solutions of 3 mg/mL pWPI or nWPI were subjected to *in vitro* digestion with pepsin (15 min) followed by trypsin, chymotrypsin and peptidase (60 min). Samples were taken every 5 min during pepsin digestion, and every 15 min during digestion with pancreatic enzymes. The protein content was determined using the Bradford method. Data are expressed as a percentage of baseline values ± SEM. Statistically significant differences in protein content between nWPI and pWPI (Tukey’s *post hoc* comparison) are designated by * *p* < 0.05.

Using a modified Bradford method for protein quantification [43,47], the protein content of the hydrolysate mixture was analyzed at different time points throughout the digestion procedure. Figure 1 shows the *in vitro* digestibility of nWPI and pWPI as assessed by protein proteolysis via the Bradford method. During the first 5 min of peptic digestion, the protein concentration decreased by 29% for pWPI, whereas the decrease was 12% for nWPI. At both the 5 and 10 min time points, the difference in DH was significant between nWPI and pWPI (*p* < 0.01), indicating that pWPI was hydrolyzed at a higher rate than nWPI. By the end of the 15 min digestion with pepsin, nWPI and pWPI showed a comparable DH (37%). During pancreatic digestion, most of the protein disappearance occurred during the first 15 min for both pWPI and nWPI. However, the DH had reached 95% for pWPI, whereas it had only reached 83% for nWPI at the 30 min time point. After 45 min of digestion, the DH was 99% and 95% for pWPI and nWPI, respectively. The difference in DH between pWPI and nWPI was statistically significant at both the 30 and 45 min time points (*p* < 0.05).

**Figure 2:**
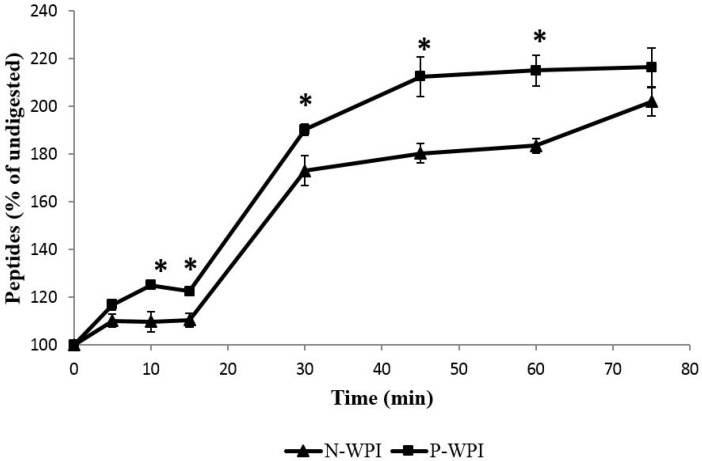
Effect of high hydrostatic pressure on *in vitro* enzymatically-driven peptide release from nWPI and pWPI. Solutions of 3 mg/mL pWPI or nWPI were subjected to *in vitro* digestion with pepsin (15 min) followed by trypsin, chymotrypsin and peptidase (60 min). Samples were taken every 5 min during pepsin digestion, and every 15 min during digestion with pancreatic enzymes. The α-amino group content was determined using the OPA reagent based on the method of Church *et al.* [44]. Data are expressed as a percentage of baseline values ± SEM. Statistically significant differences in α-amino group content between nWPI and pWPI (Tukey’s *post hoc* comparison) are designated by * *p* < 0.05.

Analysis of primary α-amino group content using the OPA reagent also showed an enhanced release of primary amines in pWPI compared to nWPI (Figure 2). During the first 5 min of pepsin hydrolysis, the rate of peptide formation was similar between pWPI and nWPI, respectively producing a 116% and 110% (*p =* 0.1) increase in peptide content relative to baseline (time = 0 min). However, peptide production rate in nWPI reached a plateau at 5 min, a slowing down which was only observed at 10 min for pWPI. The percent increase in peptide content was significantly different between pWPI and nWPI at 10 and 15 min (*p* < 0.05 and 0.01, respectively). During pancreatic digestion, peptide content was significantly higher in pWPI relative to nWPI at incubation times 30, 45, and 60 min (*p* < 0.05). At 75 min, pWPI peptide content showed a tendency to be higher than nWPI, although the difference did not reach statistical significance.

### 3.2. Effect of Hyperbaric Treatment on the Peptide Profiles of WPI Hydrolysates

Preliminary analyzes of the hydrolysates by LC-ESI-TOF-MS showed 28 peaks in nWPI hydrolysate and 31 peaks in pWPI, the majority of which were of molecular weight lower than 1000 (71.5% of nWPI and 80.6% of pWPI), suggesting that most peptides were of sizes ≤ 1 kDa (Table 1). In addition, six peptides were identified in nWPI that were no longer found in pWPI hydrolysate, and nine peptides appeared in the pWPI hydrolysate that were not found in nWPI (Table 1). 

**Table 1:** Neutral mass of peptides as assessed by LC-ESI-TOF-MS.

nWPI Hydrolysate		pWPI Hydrolysate	
Retention Time (min)	Peptide Neutral Mass ^a^	Retention Time (min)	Peptide Neutral Mass ^b^
2.827–3.048	**1043.5**		
3.251–3.387	**460.3**	3.225–3.395	**646.4**
		5.073–5.277	**700.5**
		5.395–5.565	**762.4**
5.795–5.998	672.3868	5.836–6.006	672.3878
6.218–6.541	852.4297	6.345–6.514	852.4265
		6.735–6.972	**558.3284**
			**400.7204**
6.947–7.253	700.34	7.040–7.193	700.3458
7.78–8.033	**840.5**		
7.778–8.033	512.2822	7.786–8.024	512.2812
8.033–8.236	572.3612	8.041–8.278	572.3615
8.287–8.423	803.4467	8.312–8.532	803.4456
8.728–9.067	**634.3544**	8.854–9.007	**932.5566**
9.203–9.474	860.476	9.278–9.516	860.76
9.813–10.135	1372.687	9.940–10.092	1372.879
			**813.4305**
10.729–10.898	889.4819	10.770–11.008	889.4786
11.085–11.187	695.3363	11.126–11.262	887.4665
	887.4681		695.3357
11.441–11.509	804.4482	11.4661–11.584	804.4472
11.56–11.729	452.3538	11.601–11.771	452.3443
11.882–12.085	774.4709	11.856–12.127	774.4704
		12.144–12.278	**805.4018**
12.221–12.34	1391.651	12.296–12.449	1391.876
		12.975–13.246	**884.5**
13.306–13.492	1090.635	13.314–13.551	1089.5
13.560–13.781	788.434	13.602–13.788	788.4327
13.815–14.035	782.5	13.890–14.060	782.5
14.527–14.7	678.5122	14.568–14.738	678.5115
15.035–15.239	**990.511**		
	**1099.576**		
16.460–16.578	1311.561	16.434–16.654	1311.16
17.189–17.4	1190.628	17.264–17.349	1190.623
19.292–19.4	1450.796	19.299–19.486	1450.75

^a^ Masses in bold represent peptides found in nWPI but not in pWPI hydrolysate; ^b^ Masses in bold represent peptides found in pWPI but not in nWPI hydrolysate.

**Figure 3:**
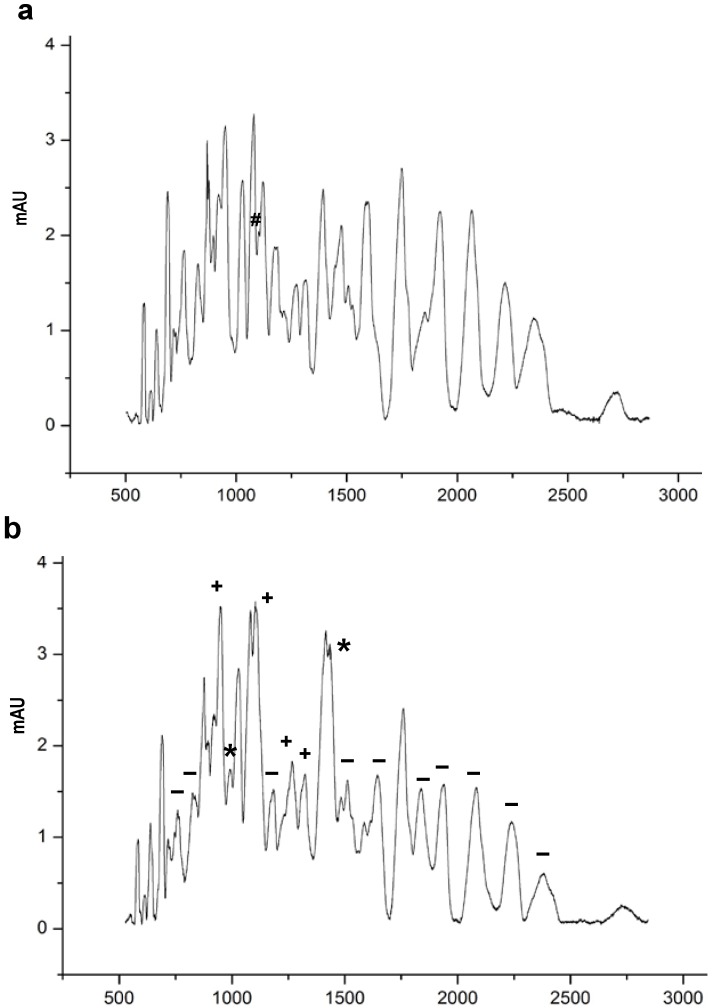
CZE profiles of peptide extracts with MWCO ≤ 1 kDa derived from pepsin, trypsin, chymotrypsin and peptidase hydrolysis of (**a**) nWPH and (**b**) pWPH. The symbols identify extra peaks found in pWPH hydrolysates (*) and indicate differences in the relative abundances of peptides as a result of pressure treatment (+, higher abundance, −, lower abundance in pWPH relative to nWPH; #, peak found in nWPH but not in pWPH hydrolysates).

**Figure 4:**
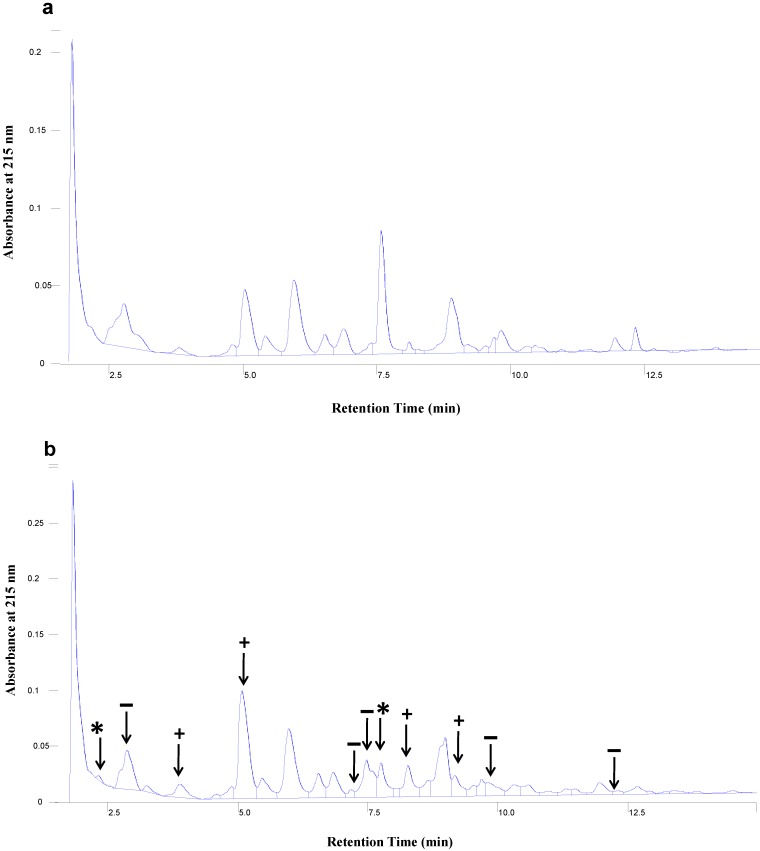
HPLC profiles of peptide extracts with MWCO ≤ 1 kDa derived from pepsin, trypsin, chymotrypsin and peptidase hydrolysis of: (**a**) nWPH and (**b**) pWPH. The arrows identify extra peaks found in pWPH hydrolysates (*) and indicate differences in the relative abundances of peptides as a result of pressure treatment (+, higher abundance, −, lower abundance in pWPH relative to nWPH).

Figure 3 shows the peptide profiles of nWPH and pWPH obtained by CZE. Lyophilized hydrolysates were reconstituted at a concentration of 2 mg/mL and separated over a 50 min elution time. The electropherograms in Figure 3 show that the peptide profiles obtained from pWPH differed from those of nWPH in terms of relative abundance. The percent area of each peak relative to the entire area under the curve was compared between pWPH and nWPH. A number of peaks were significantly increased in pWPH relative to nWPH, while others were significantly lower. In addition, two novel peaks appeared in the pWPH that were absent from nWPH, while one peak from nWPH was absent from the pWPH peptide profiles. Table 2 lists the percent areas of each peak in the nWPH and pWPH profiles. Figure 4 shows the peptide profiles of nWPH and pWPH obtained by HPLC. Here as well, differences in relative abundance were observed and two peaks appeared in pWPH that were absent from nWPH.

**Table 2:** Differences in percent peak areas between nWPI and pWPI hydrolysates, relative to the total area under the curve, as assessed by CZE.

Peak Number	% of Area Under the Curve		*p*-value
	N-WPI	P-WPI	
1	0.9100	0.7336	NS
2	0.2508	0.2149	NS
3	0.8530	0.6995	NS
4	0.0215	0.1164	NS
5	2.2934	1.8091	NS
6	0.7993	0.2026	0.0154
7	0.3922	1.1154	NS
8	2.6619	1.5572	0.027
9	2.7604	2.4542	NS
10	2.4608	2.9801	NS
11	1.4659	1.4913	NS
12	2.7565	2.4610	NS
13	4.5988	4.8079	0.046
14	0	2.0727	Only in pWPI hydrolysate
15	3.7926	4.1746	NS
16	4.2629	4.2212	NS
17	0.9752	0	Only in nWPI hydrolysate
18	3.3845	6.1931	<0.0001
19	3.7332	2.5659	0.002
20	1.3635	1.0267	NS
21	2.5261	4.2416	0.0476
22	2.6743	3.5632	0.0005
23	5.0190	5.4883	NS
24	0	4.2459	Only in pWPI hydrolysate
25	1.5836	1.5758	NS
26	3.2332	2.1505	0.0038
27	1.4856	1.1582	NS
28	1.2950	1.4485	NS
29	5.9445	3.699	0.007
30	6.8146	6.0910	NS
31	2.8035	4.1246	0.0005
32	6.0780	3.8966	0.002
33	6.3993	5.0982	0.024
34	4.7252	3.6685	0.006
35	5.3344	2.7964	0.002

### 3.3. Effect of Hyperbaric Treatment on the Ferric-Reducing Antioxidant Power of WPI Hydrolysates

**Figure 5:**
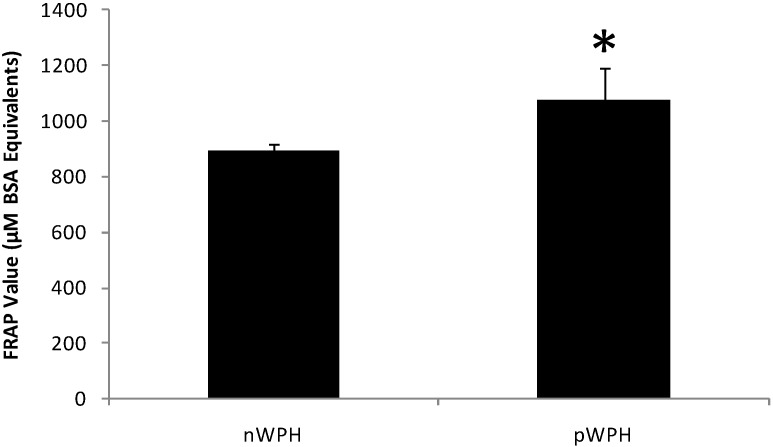
Effect of high hydrostatic pressure on the ferric-reducing antioxidant power (FRAP) of WPH. Freeze-dried hydrolysates resulting from the *in vitro* digestion and subsequent ultrafiltration were reconstituted in ddH_2_O at a concentration of 10 mg/mL and incubated with the FRAP reagent for 90 min at a 1:30 ratio. BSA was used to construct a standard curve and absorbances were read at 593 nm. Results are expressed in μM BSA equivalents ± SEM. (*) Indicates a statistically significant difference (*p* < 0.05).

Figure 5 shows the FRAP values for pWPH and nWPH. The capacity of pWPH to reduce the Fe^3+^-2,4,6-tripyridyl-*S*-triazine complex was higher than that of nWPH by 21% (*p* < 0.05).

### 3.4. Effect of Differences in In Vitro Digestion Protocols on the Antioxidant Capacity of Hydrolysates in Respiratory Epithelial Cell Culture Medium

**Figure 6:**
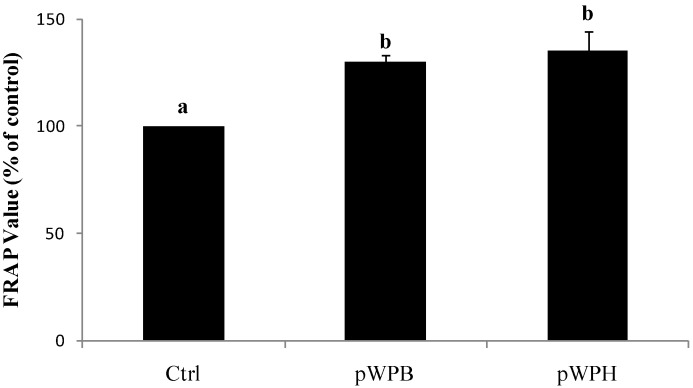
Effect of hydrolysates prepared using two different digestion protocols (pWPH (current protocol) versus pWPB (protocol of Vilela *et al*. (2006) [26]) on FRAP of cell-free supernatants. 1HAEo- cells were incubated with either pWPH or pWPB (1000 μg/mL) for 6 h and cell culture supernatant was analyzed using the FRAP assay. Data are represented as mean ± SEM of three to four independent experiments. Treatments not sharing common letters are significantly different (*p* < 0.05) by one way ANOVA and Tukey’s *post hoc* analysis.

Exposure of 1HAEo- cells to either the pWPH or the pWPB hydrolysate (1000 μg/mL) for 6 h induced an increase in FRAP by 35% and 30%, respectively (*p <* 0.05). Although the increase in FRAP following pWPH treatment was slightly higher than following pWPB treatment, the differences were not statistically significant (Figure 6).

### 3.4. Effect of Differences in In Vitro Digestion Protocols on the Inhibition of IL-8 Secretion by Hydrolysates in Respiratory Epithelial Cells

**Figure 7:**
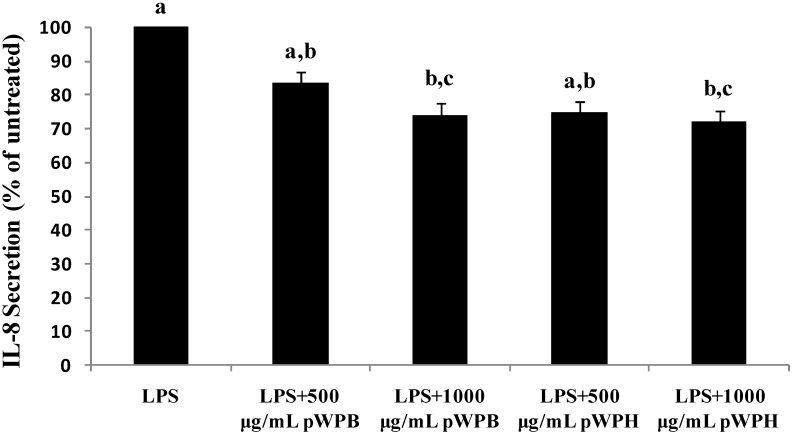
Effect of hydrolysates prepared using two different digestion protocols (pWPH (current protocol) and pWPB (protocol of Vilela *et al*. (2006) [26]) on LPS-induced IL-8 secretion. 1HAEo- cells were pre-treated with pWPH or pWPB for 1 h followed by incubation with LPS and fresh pWPH or pWPB for 24 h. Data are represented as mean ± SEM of five independent experiments. Treatments not sharing common letters are significantly different (*p* < 0.05) by one way ANOVA and Tukey’s *post hoc* analysis.

Pre-treatment of 1HAEo- cells to the pWPB hydrolysate for 6 h showed that LPS-induced IL-8 secretion was lowered at doses of 500 and 1000 μg/mL by 16% and 26%, respectively (Figure 7). Similarly, pWPH inhibited IL-8 secretion by 25% and 28% at doses of 500 and 1000 μg/mL, respectively. For both types of hydrolysates, however, statistical significance was reached only at the higher dose of 1000 μg/mL. There was no significant difference between the two types of hydrolysates in terms of IL-8 suppression. 

## 4. Discussion

This study describes a series of modifications applied to a previously described *in vitro* digestion protocol that was intended to imitate *in vivo* gastrointestinal digestion [26]. The modified *in vitro* digestive procedure and the resulting hydrolysates as described in the present study was developed to mimic physiological processes as closely as possible the *in vivo* protein digestibility and generation of bioactive peptides. The changes in digestion protocols introduced herein, including temperature, pH, enzymatic conditions and ultrafiltration method, constitute noticeable improvements in terms of further approximating human gastrointestinal digestion and absorption. The effects of such modifications on the resulting hydrolysates are illustrated by the HPLC and CZE peptide profiles from the current study, which differ markedly from those of Vilela *et al.* [26]. The modified digestion protocol employed in this study increased the peptide population by more than three-fold in comparison to the previous study. Although a number of adjustments were introduced to the current digestion procedure, the major factor responsible for the increased number of peptides was likely the use of a 10 kDa ultrafiltration membrane to isolate the peptides, as opposed to a 1 kDa membrane used in previous work. The utilization of the MWCO of 10 kDa resulted in a peptide mixture with a higher proportion of peptides relative to free amino acids and a peptide fraction containing peptides of higher and medium MW. Indeed, the LC-ESI-TOF-MS analyses showed that the peptides obtained with the use of the MWCO of 10 kDa were mostly of 1 kDa in size or smaller (Table 1), which is within the molecular weight range typically presumed to be suitable for gastrointestinal absorption [48]. 

Results from this study show that hyperbaric treatment enhanced the *in vitro* digestibility of WPI. Previous studies involving the enzymatic digestion of proteins under high hydrostatic pressure have shown accelerated reaction rates when pressure was applied during the digestion procedure [19,20,21]. However, in the context of whey proteins as functional foods, the effect of pressure-induced conformational changes exerted on the proteins prior to digestion is more relevant. To our knowledge, there are only two studies that have shown that high hydrostatic pressure pre-treatment increases the hydrolysis rate and alters the resulting peptides profiles in whey proteins, which was shown with β-LG [49] and WPI [26]. The study by Knudsen *et al*. [49] described hydrolysis experiments involving trypsin, chymotrypsin and *B. licheniformis* protease (BLP) individually for up to 240 min; however, they did not specifically investigate a simulated human gastro-intestinal enzymatic digestion. Nevertheless, following similar pressurization conditions (450 MPa), they observed an enzyme dependent percent drop in concentration of pressurized β-LG ranging from 5% (trypsin) to 15% (BLP) to 55% (chymotrypsin) after 10 min of hydrolysis, as compared to the 35% drop in protein concentration with pepsin observed in the present study. Vilela *et al.* [26] previously showed that WPI digestibility by pepsin was enhanced by pressure pretreatment, a finding confirmed herein. After 10 minutes of pepsin digestion, Vilela *et al.* [26] observed 15% drop in pWPI concentration as compared to no change in nWPI, whereas we observed a 35% drop in pWPI concentration versus a 10% decrease in nWPI. These slightly different results are likely due to differences in the digestion conditions described above, including substrate concentration and the E:S ratio. In addition, the present results extend previous work, showing that subsequent digestion by pancreatic enzymes is also enhanced. The process of pressurization likely helped to expose cleavage sites within the proteins that would be otherwise unavailable to the action of pepsin. The resulting improved digestibility by pepsin can lead to the formation of intermediate polypeptides that are, in turn, more readily hydrolyzed by pancreatic enzymes. Variations in hydrolysis methods such as the choice of enzymes, the E:S ratio, temperature and pH can result in hydrolysates with different peptide profiles and their biological activities can markedly differ in nature or in magnitude [27,28]. We have previously demonstrated that pre-incubation of respiratory epithelial cells with pWPH led to inhibition of LPS-induced IL-8 secretion and increased cell-culture medium FRAP [29]. Hence, in the present study we compared the IL-8 suppressing and FRAP-enhancing capacities of the hydrolysates produced via these two different digestion methods, which were found to be within the ranges observed previously [29]. Although the bioactivity of pWPH was slightly more potent than that of pWPB in both experiments, the differences were not statistically significant. Therefore, the modifications to the digestion procedure implemented herein, while allowing for a better approximation of *in vivo* digestion, do not significantly affect the observed biological activities of the hydrolysates.

Enzymatic hydrolysis of proteins is often used to produce hydrolysates with functional or bioactive properties, or to investigate the digestibility and bioavailability of particular proteins. Whey proteins are globular molecules with organized secondary and tertiary structures stabilized by disulfide (S–S) bonds. β-LG, α-LA and BSA contain two, four and seventeen S–S bonds, respectively [50]. These features help render most whey proteins relatively resistant to proteolytic hydrolysis by enzymes such as pepsin, trypsin or chymotrypsin [51]. The primary sequence of β-LG, for example, contains peptide bonds that can be cleaved by trypsin or pepsin, but are located within the protein’s hydrophobic core and therefore inaccessible to the enzyme [49,52]. In addition, being soluble at acidic pH, whey proteins exhibit a relatively shorter gastric emptying time and exit the stomach after a shorter exposure to peptic hydrolysis relative to other dietary proteins. In view of the above and in consideration of the significant functional effects of whey proteins observed in animal and clinical studies, enhancing the susceptibility of these proteins to gastrointestinal digestion via pressurization could potentially increase the bioavailability of bioactive peptides present in their primary sequence.

Many of the beneficial effects of whey proteins are attributed to their ability to afford protection against oxidative stress. In animals, whey protein feeding has shown antioxidative effects that have been related to upregulation of antioxidative enzymes such as superoxide dismutase, catalase and glutathione peroxidase [53] and decreased tissue concentrations of free radical oxidation products [54]. In addition to being rich in branched chain amino acids, whey proteins are notable for their high content of sulphur-containing amino acids (cysteine, methionine) [55]. Cysteine is a rate-limiting precursor for the biosynthesis of glutathione (l-γ-glutamyl-l-cysteinyl-glycine), a ubiquitous tripeptide thiol reducing agent. In addition to their glutathione-enhancing effects, amino acids and peptides derived from the hydrolysis of whey proteins have been shown to have antioxidant properties. Peptides from β-LG and α-lactalbumin exhibit free-radical scavenging activity *in vitro* [56]. Whey peptides or hydrolysates also inhibit iron-catalyzed lipid oxidation in liposomes [57,58,59]. The antioxidant effects of these peptides could be mediated by particular peptide structures or via specific side-chain groups in amino acid residues [60,61]. It has been recently determined that acidic amino acids (glutamic acid and aspartic acid) are strong contributors to the FRAP of food protein hydrolysates, due to their hydrogen-donating ability [62]. Sulphur-containing amino acids (cysteine and methionine) are the most potent at reducing the Fe^3+^-2,4,6-tripyridyl-*S*-triazine complex owing to their sulfhydryl group [62]. In light of this fact, and that l-γ-glutamyl-l-cysteine is a rate-limiting precursor for GSH synthesis, this could be a partial explanation for the relatively higher rate of glutathione accumulation in lymphocytes of healthy subjects after WPI supplementation observed in previous studies [23].

Our results also show that hydrolysates from pWPH had a significantly higher FRAP values than nWPH. The α-amino group content of pWPH hydrolysates was not significantly different from that of nWPH at the end of the digestion. Therefore, the increased antioxidant power was not attributable to an increase in peptide concentration but rather could be related to a relative increase in the abundance of antioxidant peptides and amino acids in pWPH. It is likely that the process of pressurization exposed particular cleavage sites within the whey proteins, affording the proteolytic enzymes better access to more readily produce specific peptide sequences containing these amino acids. Hence, it is conceivable that the observed quantitative and qualitative differences in peptide profiles induced by pressure treatment reflect an enrichment of the final mixture with specific peptides possessing antioxidant activity. In food systems, the antioxidant properties of whey protein-derived peptides are well documented, particularly in iron-catalyzed liposomal systems [57,58], suggesting their value in preventing or slowing lipid and fatty acid peroxidation in foods. The increased FRAP of pressurized in comparison to native whey protein isolate hydrolysates indicates new possibilities for the application of high hydrostatic pressure to enhance antioxidant properties of whey peptides for use as natural functional ingredient in food products.

Several studies have shown that high hydrostatic pressure pretreatment of whey proteins increases their hydrolysis rate [26] and alters the resulting peptide profiles of β-LG [52]. In the study by Vilela *et al.*, a two-stage *in vitro* digestion protocol was adapted to mimic human gastrointestinal digestion and absorption. The nWPI and pWPI were first subjected to hydrolysis by pepsin at acidic pH, followed by pancreatin at near-neutral pH and a final ultrafiltration step through a 1 kDa membrane to isolate low molecular weight peptides. It has been shown that peptides of sizes up to 1 kDa can be absorbed through the intestinal lumen via di- and tri-peptide transporters or paracellular pathways [48,63,64] in quantitatively significant amounts to exert their bioactive effects. It is possible that the peptide mixture obtained via the digestion procedure of Vilela *et al.*, may have quantitatively and qualitatively underestimated the generation of peptides under 1 kDa from pWPI, as the MWCO of ultrafiltration membranes are based on their ability to retain molecules of a given size. Several peptide species may have therefore been eliminated from the final permeate.

At the end of the stage 2 pancreatic digestion, both pWPH and nWPH solutions had undetectable protein levels as assessed by the Bradford assay. The Bradford assay cannot detect polypeptides with sizes below 3 kDa, indicating that the final hydrolysate mixture consisted chiefly of small polypeptides and amino acids. It is noteworthy that during the digestion procedure the protein concentration of pWPH reached zero after 60 min, which was notably faster than nWPH. At times 30 and 45 min, there remained respectively 4-fold and 14-fold more protein in the nWPH solution as compared to pWPH, which indicates that proteins and peptides larger than 3 kDa disappear faster during pWPI digestion. It is likely that an increased rate of appearance of small peptides *in vivo* is accompanied by an increase in their rate of absorption. It has been shown that the rate of absorption of amino acids from dietary proteins influences whole body protein deposition by affecting the breakdown, synthesis and oxidation of proteins [36]. As leucine balance can be used as an index of whole body protein deposition [65], the postprandial rise in plasma amino acids has been examined in healthy subjects using l-[1-^13^C]leucine-labeled casein or whey protein [36]. It was found that the ingestion of whey protein resulted in greater and more rapid transient increases in plasma amino acid levels than casein intake, which was related to an augmented rate of protein synthesis and oxidation as compared to casein. However, protein breakdown was inhibited by casein whereas it was unaffected by whey protein ingestion, although the addition of energy in the form of carbohydrate did induce inhibition of proteolysis [36]. In contrast, elderly subjects given a whey protein meal exhibited higher postprandial protein utilization efficiency than after a meal with casein, as well as a higher postprandial leucine balance [66,67]. Although a slow release of amino acids into the circulation is desirable in some conditions such as hepatic encephalopathy or renal insufficiency [68], strong hyperaminoacedemia could be beneficial for the elderly, as it can reverse the impaired muscle protein synthesis following feeding [69,70]. The resulting improvement in protein synthesis could be advantageous in limiting muscle protein loss in the elderly. Other implications of such differences in postprandial aminoacidemia are illustrated by findings in patients with Type 2 diabetes mellitus, as a mixed meal containing whey protein resulted in a greater beta-cell response than a casein meal, as assessed by levels of insulin, pro-insulin and C-peptide. The differences in beta-cell responses were attributable to the proteins’ digestion and absorption pattern rather than their amino acid composition, as the administration of a meal containing casein-like free amino acids elicited similar responses to those of whey protein [71]. A greater rise in plasma amino acids was also observed in conjunction with increased satiety after whey protein ingestion in comparison to casein, suggesting its usefulness in weight management and the treatment of obesity [72]. All of the above postprandial effects of whey proteins were attributed to their more rapid gastric emptying leading to a more pronounced appearance of amino acids in the circulation as opposed to casein, a “slower” protein. These findings, in conjunction with the increased *in vitro* digestibility of pWPI observed herein, suggest that pressurization of whey proteins could potentiate the observed beneficial effects on protein metabolism described above.

## 5. Conclusions

In summary, we have described a series of modifications to a previous *in vitro* digestion protocol that better approximates *in vivo* digestion, producing hydrolysates with an increased peptide population and different peptide profiles. We have also demonstrated that, even when different digestion procedures are used, pressurization of whey proteins improves their *in vitro* digestibility by pepsin and pancreatic enzymes, and modifies the resulting peptide profiles. In addition, these modifications were not shown to significantly affect the enhanced capability of hydrolysates from pressurized WPI over native WPI towards their IL-8 suppressing and FRAP enhancing abilities in cultured 1HAEo- cells. Additionally, the present findings demonstrate that pressurization of whey proteins enhances their *in vitro* antioxidant capacity. These *in vitro* studies suggest that high hydrostatic pressure treatment offers promising prospects for the modification of food proteins leading to functional enhancements of their bioactive properties.
